# Determinants Associated With Obesity in Children of Low Socioeconomic Status Families: A Narrative Review

**DOI:** 10.1155/jobe/4992624

**Published:** 2025-09-27

**Authors:** Zeena Harakeh, Wilma Otten, Pepijn van Empelen

**Affiliations:** ^1^Department of Child Health, TNO, Netherlands Organization for Applied Scientific Research, Leiden, the Netherlands; ^2^Department of Sustainable Productivity and Employability, TNO, Netherlands Organization for Applied Scientific Research, Leiden, the Netherlands

**Keywords:** children, eating, environmental determinants, individual determinants, obesity, physical activity, review, sedentary behavior, socioeconomic status

## Abstract

Children of families with low socioeconomic status (SES) are at higher risk for obesity and obesity-related lifestyle behaviors, i.e., unhealthy eating and low physical activity. This review aims to identify changeable determinants of obesity and obesity-related lifestyle behaviors in children aged 0–12, with a focus on those specific to low SES. A literature search was conducted in PsycINFO/Ovid and PubMed, using terms related to SES, obesity, and individual or environmental determinants. We included 42 systematic review/meta-analysis articles, written in English, that focused on children (0–12 years) and assessed obesity or obesity-related lifestyle behavior outcomes. We extracted modifiable individual and environmental determinants, and the role of SES in their association with obesity and obesity-related lifestyle behaviors in children. Nine reviews examined the relationship between determinants and obesity and obesity-related lifestyle behaviors in children, and the role of SES. These reviews focused mainly on environmental determinants (*n* = 8), particularly family and peer factors (*n* = 6). The findings suggest that SES may influence obesity and lifestyle behaviors indirectly through parental factors, such as parental BMI, maternal smoking during pregnancy, and parental TV viewing behaviors. SES may also moderate the impact of parental factors, such as parental BMI, maternal depression, or permissive/indulgent parenting. Our review showed that research on determinants of obesity and obesity-related lifestyle behaviors of children with low SES is limited, with scarce and inconsistent evidence and lacking theoretical explanations. The (parent-related) mechanisms which influence child obesity in families with low SES are still unclear. To develop effective (family) interventions to prevent or decrease obesity in children of families with low SES, future research needs to examine individual and environmental determinants and underlying mechanisms through which SES has its influence on childhood obesity.

## 1. Introduction

Obesity and obesity-related lifestyle behaviors start early in life [1–3]. Therefore, in order to prevent obesity later in life, childhood is an important period to focus on. Obesity in children has adverse consequences regarding physical and psychological health [4] and is associated with obesity in adulthood. Being obese or overweight results from an imbalance between energy intake and energy expenditure due to an unhealthy eating lifestyle (including high-calorie/low-nutrient foods and beverages) and low physical activity (PA) (including engagement in sedentary activities) [5]. Some eating behaviors have shown to be risk factors (e.g., sugar-sweetened beverages, eating diets high in animal products, high protein intake, total calorie intake, eating take-out foods, fast-food consumption, high number of servings of snacks per day), whereas others have shown to be protective factors (e.g., eating fruits and vegetables, dietary restraint behaviors) of obesity in children [6, 7]. Furthermore, low PA and sedentary behaviors (e.g., television viewing, computer use, video gaming) have been shown to be risk factors of obesity in children [6–8].

**Figure 1:**
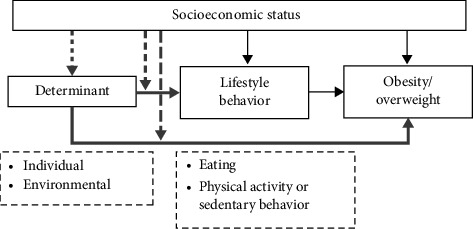
The mechanisms through which SES may influence a child's weight.

Obesity and an unhealthy lifestyle are more prevalent among children of families with low socioeconomic status (SES) [7, 9–12]. Recent literature highlights possible mechanisms linking low SES to childhood obesity via unhealthy dietary and PA patterns. Children of families with a low SES are more often exposed to unhealthy food and built environments, limiting access to nutritious food and (safe) spaces for PA. These exposures may be shaped by daily living conditions such as financial stress, poor housing, and long working hours, which reduce the capacity for healthy choices. Moreover, inequities in income and employment may affect the affordability of healthy food and organized sports, while the convenience and lower cost of unhealthy food may further drive unhealthy behaviors [13, 14]. To develop effective interventions focusing on reducing obesity in children of families with low SES, we need to gain insights into the modifiable determinants that are associated with obesity and obesity-related lifestyle behaviors, and whether there are unique determinants for children of families with low SES. There are several socioecological models (e.g., social ecological theory [15]; ecological systems theory [16]) and obesity frameworks (e.g., the Environmental Research Framework for Weight Gain Prevention (EnRG) [17]; the socioecological model adjusted to pediatric obesity [18]) that assume that both individual and environmental determinants play a role (e.g., [15]). Based on these models/frameworks (including theory of planned behavior [19]), individual determinants comprise social–cognitive and affective factors: that is, attitude, self-efficacy, subjective norm, intention, self-esteem/depression, self-control/self-regulation, temperamental negative reactivity, and stress. Furthermore, these models and frameworks (including the Analysis Grid for Environments Linked to Obesity (ANGELO) [20]) indicate that environmental determinants can be categorized into different levels of the environment: that is, interpersonal environment (i.e., family and peers such as friends and siblings), organizational environment (i.e., school, physical home), community environment (i.e., neighborhood), and societal environment (i.e., political and cultural environment) [16, 21]. Moreover, the Precede phase in the Precede-Proceed model [22] also involves the identification of individual and environmental determinants in order to develop an intervention/program that targets a specific health problem. In addition, a system approach is needed (including a multideterminant perspective and different stakeholders) in interventions/programs to prevent and reduce childhood obesity [14, 23]. Based on these frameworks and models, we depicted in Figure 1 a theoretical framework on how these determinants could be associated with obesity/overweight and obesity-related lifestyle behaviors (i.e., eating behavior, PA, and sedentary behavior) in children. One possible mechanism is an indirect relation, in which SES is associated with these determinants which, in turn, are associated with obesity and/or lifestyle behaviors (see dotted arrow in Figure 1). Another possibility is a moderation effect, in which SES moderates the association between these determinants and obesity and/or lifestyle behaviors (see dashed arrows in Figure 1).

The aim of this narrative review is to identify which changeable determinants are associated with obesity and obesity-related lifestyle behaviors in children (0–12 years), which are unique determinants for children of families with low SES. This will provide insights into which changeable determinants to target in interventions for reducing obesity in children with low SES. The following two research questions will be addressed in this review: (1) which changeable individual determinants are associated with obesity and obesity-related lifestyle behaviors in children, and the role of SES in this association (i.e., indirect relation and/or moderation effect); (2) which changeable environmental determinants are associated with obesity and obesity-related lifestyle behaviors in children, and the role of SES in this association (see the gray arrows in Figure 1).

## 2. Materials and Methods

### 2.1. Search Strategy

For this study, there is no review protocol nor was it registered. Furthermore, we have applied the reporting guidelines for narrative reviews: i.e., Scale for the Assessment of Narrative Review Articles (SANRA) (see also the filled-in narrative review checklists in Appendix 1). We performed a search of articles in PsycINFO/Ovid and PubMed databases in February–April 2020. We restricted our search including systematic reviews or meta-analyses, focusing on the childhood period (i.e., birth to 12 years) and those that were published in English. We used search terms related to SES, obesity, and individual or environmental determinants. We combined SES search terms (i.e., {social status OR socio-economic status OR socioeconomic education OR Social Class OR Socioeconomic Factors OR family affluence OR Digital Divide OR Health Status Disparities OR Educational Status OR SES OR Schooling attainment OR family income OR low income OR socioeconomic position OR socioeconomic level OR economic level OR assets}) and obesity search terms (i.e., {BMI OR Body Mass Index OR obesity OR obese OR overweight}). Also, we combined these search terms with search terms of individual factors (i.e., psychosocial, psychological, individual, Theory of Planned Behavio(u)r, self-efficacy, self-esteem, attitude, social norm, self-control, self-regulation, depress^∗^, motivation, intention) or environmental factors (i.e., {peer^∗^ OR friend^∗^ OR sibling}, environment^∗^, cultur^∗^, school, community, neighbo(u)rhood, {social OR societal OR food price OR marketing}). Additionally, we also combined the search terms socioeconomic and obesity and child and one determinant (i.e., network, family, psychosocial, psychological, individual, Theory of Planned Behavior(u)r, attitude^∗^).

In September 2020, we extended the literature search for obesity-related lifestyle behaviors with PA (including sedentary behavior) (i.e., {sedentary behavio^∗^ OR energy expenditure OR exercise}) or dietary/eating behavior (i.e., {eat^∗^ OR diet^∗^ OR food OR nutri^∗^ or energy intake OR calorie intake}). However, this resulted in no additional inclusion of systematic reviews/meta-analyses: 3 articles fitted the inclusion criteria but were found in the earlier search and therefore redundant. In March 2025, we updated the search for obesity as well as obesity-related lifestyle behaviors. We applied Automated Systematic Review (ASReview), which is an open-source tool that uses machine learning to accelerate and support the process of systematic literature screening [24]. We checked with ASReview the titles and abstracts of 25% of the total articles retrieved by the literature search, of which 3 articles fitted the inclusion criteria.

### 2.2. Inclusion and Exclusion Criteria

The inclusion criteria included a human sample, a review/meta-analysis, focusing on childhood (0–12 years) obesity, and written in English. It is important to note that studies that did not have obesity as the main outcome were still eligible for inclusion if they included obesity-related lifestyle behavior (i.e., eating behavior, PA, or sedentary behavior).

### 2.3. Study Selection and Data Extraction

Two of the authors (Z.H. and W.O.) screened titles and abstracts using the inclusion and exclusion criteria. We extracted the modifiable individual and environmental determinants to describe the results of the association of these determinants with childhood obesity (including obesity-related lifestyle behaviors). We categorized the extracted environmental determinants into different levels of the environment, i.e., interpersonal environment, organizational environment, community environment, and societal environment [16, 21]. In addition, we also extracted the role of SES (i.e., indirect relation and/or moderation effect) to describe specifically the results of the systematic reviews/meta-analyses that focused on the role of SES in the association between determinants and childhood obesity (including obesity-related lifestyle behaviors).

## 3. Results

### 3.1. Description of Reviews

We selected 42 systematic reviews/meta-analysis which focused on determinants of children's obesity and obesity-related lifestyle behaviors at the individual/child (social–cognitive and affective factors; *n* = 7), interpersonal (family and peers; *n* = 23), organizational (school; *n* = 3 and physical home *n* = 3), community (neighborhood; *n* = 6), and societal (cultural environment *n* = 2 and policy *n* = 1) level (for more information about these reviews, see Table 1). The number of studies analyzed within a systematic review/meta-analysis sometimes varied for each included determinant in this specific review/meta-analysis. Some of these reviews included both children and adolescents. In total, nine reviews focused on the association between determinants and children's obesity and obesity-related lifestyle behaviors, and the role of SES. These nine reviews focused mainly on environmental determinants (*n* = 8), specifically at the interpersonal level (*n* = 6).

### 3.2. Results of Individual Determinants

The results of the individual determinants (i.e., social–cognitive and affective factors) are depicted in Table 2. With regard to **child obesity/overweight,** the following determinants were identified. Self-regulation factors (including inhibitory control, delay of gratification, self-control, emotion regulation) showed to be protective factors [25, 26], whereas temperamental negative reactivity (including negative mood, difficult temperament, emotionality, and fear) showed to be a risk factor [26]. Several types of low self-esteem were measured; global self-esteem showed to be a risk factor for obesity [27], whereas mixed evidence was found for physical appearance self-esteem [27]. Furthermore, a mediation/indirect relation might explain how SES plays a role in the association between self-regulation and overweight in children [28]. Poverty was related to lower self-regulation, which in turn was related to more weight in children [28], and, in addition, poverty was directly associated with more weight in children.

With regard to **child's lifestyle behaviors**, the following determinants were identified. Intentions such as to engage in future health-related behaviors or to be active [29, 30] and PA preferences [30] showed to be protective factors for child's PA. Also, intention to eat healthy, preferences, and liking healthy food showed to be protective factors for child's healthy eating behavior (more consumption of fruit, fruit juice, and/or vegetables), whereas intention to consume sugar showed to be a risk factor of child's healthy eating behavior (more sugar snacking) [31]. Furthermore, for child's **PA**, perceived barriers were shown as a risk factor [30], whereas mixed evidence was found for self-efficacy and attitudes [30].

### 3.3. Results of Environmental Determinants

The results of the environmental determinants are depicted in Table 3. Environmental determinants were categorized into interpersonal level (i.e., family, peers), organizational level (physical home, school), community level (neighborhood), and societal level (i.e., culture, policy).

#### 3.3.1. Interpersonal Level

##### 3.3.1.1. Family/Parents

With regard to **child obesity/overweight**, the following determinants were identified. Protective factors were an authoritative parenting style (refers to general parenting in which parents' style include high demandingness and high responsiveness [32–35]), authoritative feeding style (refers to an authoritative parenting style specific to feeding or eating interactions [33, 35]), and pressure to eat (refers to parenting practices to encourage or persuade their child to eat enough food [33]). Risk factors were parental BMI [36, 37], maternal smoking during pregnancy [38], mother's (chronic) depression [39, 40], poor family functioning (including family member's roles, communication structures and affect regulation [41]), authoritarian parenting style (refers to general parenting in which parents' style include high demandingness and low responsiveness [35]), permissive/indulgent parenting style (refers to general parenting in which parents' style include low demandingness and high responsiveness [35]), indulgent feeding style (refers to an indulgent parenting style specific to feeding or eating interactions [33, 35]), and restrictive/controlling feeding [33, 42, 43]. Inconsistent findings were found for frequency of family meals, modeling/support of food intake, PA or media use by caregiver, and rules around media use by caregiver [44–46].

Furthermore, indirect relations might explain how SES plays a role in the association between parental BMI, maternal smoking during pregnancy on the one hand, and overweight in children on the other hand. Lower SES is related to a higher parental BMI, which in turn is related to more adiposity/overweight in children [36, 37]. However, the evidence for maternal smoking during pregnancy was inconsistent. Oken and colleagues [38] and Mech and colleagues [36] showed that there were no indirect relations between SES and child's overweight via smoking during pregnancy and current smoking, whereas Gebremariam and colleagues [37] did show that lower SES was related to maternal smoking during pregnancy, which in turn was related to more adiposity in children.

In addition, a moderation effect might explain how SES plays a role in the association between parental BMI, maternal depression, and permissive/indulgent parenting style on the one hand and overweight in children on the other hand. Mech and colleagues [36] showed, besides an indirect relation, also a moderating effect for parental BMI: for families with low SES, parental BMI increased child's overweight. However, the evidence for maternal depression was inconsistent. Lampard and colleagues [39] did not show a moderating effect of income on mother's depression and child's overweight, but Mech and colleagues [36] showed that low SES reinforces the relation between mother's depression and child's weight. Mech and colleagues [36] showed a moderating effect of a permissive parenting style: For high SES families, a permissive parenting style was a risk factor for a child's overweight.

With regard to **child's lifestyle behaviors**, the following determinants were identified. An authoritative parenting style [32, 34, 35] showed to be a protective factor for eating behavior, whereas inconsistent findings were found for PA [34, 35]. Furthermore, protective factors for child's **PA** were shown for parental beliefs that participating in PA is important, parents' positive attitudes toward PA, parent's encouraging PA, parents' setting rules, parents providing transportation, paying fees and tuition, parents' engaging in PA activities with children [11, 47], parent's PA (i.e., modeling [11, 47, 48]), and parental support [47, 48]. A risk factor for child's PA was shown for parental concerns about safety (including concerns related to neighborhood and community safety such as crime and traffic [11]). In other words, higher parental concerns were related to lower PA.

Protective factors for **eating behavior** were shown for availability of healthy foods [49], nonavailability of unhealthy foods [49], parental control of availability of unhealthy foods [49, 50], frequency in which parents eat healthily (i.e., parental modeling [49]), restrictive/controlling feeding [43], and norms regarding healthy eating [31]. Furthermore, risk factors for child's eating behavior were shown for parental restriction of food [43, 50], availability of unhealthy foods [50], and norms regarding unhealthy eating [31]. Mixed evidence regarding eating behavior was found for pressure to eat, monitoring, and positive parent behaviors (i.e., role modeling and rules about eating [50]). Moreover, McClain and colleagues [31] showed that modeling regarding healthy eating was a protective factor and modeling regarding unhealthy eating a risk factor, but only when reported by the child, whereas parent reports showed mixed evidence.

With regard to **sedentary screen behavior**, indirect relations were shown for SES. Lower SES was related to having a TV in the child's bedroom, parental modeling for TV viewing, parental co-viewing, and eating meals in front of the TV, which all contributed to sedentary screen behavior [51].

##### 3.3.1.2. Peers

With regard to **child's lifestyle behaviors**, the following determinants were identified. Protective factors for child's **PA** were shown for encouragement from friends (i.e., communicating social norms), friends' own PA (i.e., modeling), and engagement with friends in PA [52]. Protective factors for **eating behavior** were shown for friends' concern for eating healthy food, peer approval, perceived engagement level of siblings, and (descriptive) norms [31, 53]. Furthermore, risk factors for child's eating behavior were shown for peer support for unhealthy eating, adaptation to the eating habits of peers, peers' liking of unhealthy food, and eating out with peers [53].

#### 3.3.2. Organizational Level

##### 3.3.2.1. Physical Home Determinants

With regard to **child obesity/overweight**, the following risk factor was identified: having media equipment (e.g., TV, computer, mobile phone) available and accessible in the home and/or in the bedroom [46]. Mixed evidence regarding obesity/overweight was found for availability and accessibility of food or PA equipment in the home [46].

With regard to **child's lifestyle behaviors**, the following home environmental determinants were identified. Using prominent exergaming materials (i.e., exergaming bike, dance mats), the presence of a backyard and PA materials [54] showed to be protective factors for child's PA. Furthermore, mixed evidence regarding PA was found for active videogames [55]. TV limiting devices [54, 55] showed to be a protective factor for child's sedentary behavior. Furthermore, risk factor for child's sedentary behavior was shown for having media equipment (e.g., TV, computer, mobile phone) in the home and/or in the bedroom [54, 55]. Mixed evidence regarding sedentary behavior was found for PA equipment [55].

##### 3.3.2.2. School Determinants

With regard to **child obesity/overweight**, the following determinants were identified. Protective factors were portable and fixed play equipment, indoor space for active play, more time of free play outdoor and indoor, no high sugar and high fat food served having healthy weighed educators and active educators [56], minutes of recess and physical education, meeting recommended recess and physical education time, percentage of parental involvement in school, healthful school food environment, and school's SES index score [57]. Risk factors were sedentary opportunities (e.g., seated activities, TV viewing, and video game playing [56]), a lack of nutrition and PA policies, a lack of providers knowing effective nutrition and physical activities, a lack of adequate equipment and space for indoor and outdoor playtime activities, misperceptions regarding nutrition among providers, poor nutrition-related communication with families, and poor feeding practices [58].

#### 3.3.3. Community Level

##### 3.3.3.1. Neighborhood Determinants

With regard to **child obesity/overweight**, the following determinants were identified. School play space, road safety, proximity to supermarkets, lower population density [59], and neighborhood economic status [60] showed to be protective factors. SES showed to play a role as moderator for neighborhood hazards (e.g., litter, trash, noise [59]). More hazards were related to a lower children's BMI for low SES, whereas this relation was not found for high SES.

With regard to **child's lifestyle behaviors**, the following determinants were identified. Publicly provided recreational infrastructure (i.e., access to recreational facilities and schools), publicly provided transport infrastructure (i.e., presence of sidewalks and controlled intersections, access to destinations and public transportation) [61, 62], and age appropriate outdoor play spaces with access to play equipment [11, 62] showed to be protective factors for child's PA. Furthermore, risk factors for child's **PA** were shown for transport infrastructure (i.e., number of roads to cross and traffic density/speed), local socioeconomic conditions (i.e., crime, area deprivation, e.g., rates of car ownership, housing tenure, unemployment, and overcrowding in the district) [61], and perceived lack of neighborhood safety [11]. Furthermore, SES did not play a role in the relationship between built environment and PA [63]. There was no support for an indirect relation between SES and PA via built environment. Also, SES did not seem to be a moderator in the relationship between built environment and PA, except for mixed findings regarding accessibility of the built environment.

#### 3.3.4. Societal Level

##### 3.3.4.1. Cultural Determinants

Mixed evidence regarding obesity, PA, and eating behavior was found for acculturation [11, 64].

##### 3.3.4.2. Policy Determinants

A protective factor for overweight/obesity was found for policies to regulate competitive foods and beverages (e.g., candy, chips, and sodas) sold in schools [65].

## 4. Discussion

In general, children of families with a low SES are more obese, eat more energy rich food/drinks, and are physically less active than children of families with a high SES [7, 9–12]. In this review, we aimed to identify which changeable determinants could explain children's obesity and obesity-related lifestyle behaviors, and in particular, which are specific for children (i.e., 0–12 years) with low SES. Given the abundance of research in this domain, we focused on systematic reviews and meta-analysis (*n* = 42). We did indeed find a large variety of individual and environmental determinants that affected obesity and lifestyle behaviors. The majority of the systematic reviews/meta-analysis we selected focused on determinants of children's obesity and obesity-related lifestyle behaviors at the interpersonal level (*n* = 23), especially on parental determinants discussed in these reviews. However, the majority of these reviews did not specifically focus on whether these (parental) determinants differed for children from low SES and high SES families.

In total, we included nine reviews that focused on whether the determinants differed for children from low SES and high SES families; specifically, six of these reviews focused on parental determinants. These results suggest that SES may influence obesity and lifestyle behaviors indirectly through parental factors, such as parental BMI, maternal smoking during pregnancy, and parental TV viewing behaviors [36, 37, 51]. SES may also moderate the impact of parental factors, such as parental BMI, maternal depression, or permissive/indulgent parenting [36]. These results provided sometimes inconsistent evidence such as for smoking during pregnancy and maternal depression. One possible explanation for this inconsistency of findings is that the operationalization of these determinants matters. For example, the findings of Lampard and colleagues [39] showed an association between maternal chronic depression (i.e., multiple times depression) and child's overweight, but not for maternal episodic depression (i.e., single time depression). Also, the operationalization of SES, which is of a multifaceted nature, might have contributed to the inconsistency of findings in the literature. For example, parental education seems to be a stronger predictor for obesity than parental occupation or income [9]. Furthermore, the underlying theoretical explanations/mechanisms to explain these results were lacking. For example, Oken and colleagues [38] discussed that nicotine during pregnancy might impact later child's health outcomes because of the sensitivity of the prenatal period, although they also indicated that the exact mechanism is not clear. Lampard and colleagues [39] suggested that the association between maternal depression and child's overweight could be explained through the impact of maternal depression on parenting practices, like child diet, screen behaviors, and PA. In addition, the underlying theoretical explanations/mechanisms through which SES has its influence on child obesity also might differ for each SES indicator (Sobal, 1991 in [9]).

Two other systematic reviews/meta-analysis showed that also individual (i.e., self-regulation) and neighborhood (i.e., neighborhood hazards) determinants differed for children from low SES and high SES families. Hails and colleagues [28] showed that poverty was related to lower self-regulation, which in turn was related to more weight in children. Self-regulation was conceptualized as “hot” regulation (e.g., delay of gratification), “cold” regulation (e.g., inhibitory control), and self-regulation of appetite. Spruijt-Metz [8] mentions that low SES children are more prone to eat unhealthily due to a stress response, because they live in more stress-arousing situations. Dunton and colleagues [59] showed that more neighborhood hazards (e.g., litter, trash, noise) were related to a lower children's BMI for low SES, whereas this relation was absent for high SES. For the remaining environmental determinants, that is, physical home, school, and societal determinants, no moderating and/or mediating effects of SES were found. However, schools with higher SES had less obese pupils in the long run [57]. Also, a higher neighborhood economic status was consistently related to less obesity in children [60], but no conclusive results were obtained for eating and PA. Davison and Lawson [61] showed that children's PA was lower in neighborhoods with crime or area deprivation (e.g., rates of car ownership, housing tenure, unemployment, and overcrowding in the district), suggesting lower SES neighborhoods.

## 5. Limitations and Future Research

This review has some limitations. First, the literature search was initially conducted till September 2020 and then updated till March 2025. The search was limited to the two databases PsycINFO/Ovid and PubMed. This might have resulted in missing out on some recent systematic reviews/meta-analyses or recently published empirical research articles that examined determinants of obesity and obesity-related lifestyle behaviors among children (0–12 years), specific for low SES. To ensure that we did not miss out on recently published empirical research articles in the initial search, we did the search for publication year [2019–2020]. This resulted in six individual empirical articles [66–71]. The results of the studies of Hidalgo-Mendez and colleagues [67] and Pesch and colleagues [66] showed that the effect of SES on the relation between an indulgent, permissive feeding style is a risk factor for obesity in high SES families. This is in line with the result of the systematic review of Mech and colleagues [36], which showed that an indulgent, permissive feeding style is a risk factor for obesity in high SES families. Furthermore, Coto and colleagues [68] observed the effect of feeding practices, like the availability of fruits and vegetables and parents being a healthy role model promoting healthy eating, in low SES families, but their results are inconclusive because it was not compared to a high SES group. We decided not to include these six individual empirical articles in our review as these studies all used a cross-sectional design. In addition, future research should conduct more longitudinal and experimental studies. Although existing systematic reviews/meta-analyses did incorporate these type of research designs, the majority of these existing reviews and the recent individual empirical articles conducted cross-sectional studies, and thus, cause–effect conclusions cannot be drawn from these findings.

Second, some of the included systematic review studies/meta-analyses did not distinguish between children and adolescents, which makes it unclear whether the associations between the determinants on the one hand and children's obesity and obesity-related lifestyle behaviors on the other hand might differ for the different age groups. Also, the age group, 0–12 years, included in this review might need to be split up into different age groups/phases to identify possible specific determinants. Moreover, the results might also differ for boys and girls.

Third, we based the direction of the association on what the authors of the included systematic reviews/meta-analyses concluded. However, some reviews did not seem to use criteria to conclude whether the findings were consistent or mixed, whereas others did (e.g., if the number of positive associations over the articles exceeded 50% they considered it a consistent relation [31]).

Finally, the inconsistency of findings in the literature might be due to the operationalization of SES, the determinants, and/or children's obesity and obesity-related lifestyle behaviors. Not only SES is multifaceted, so are eating and PA as described in the introduction. Additionally, also the various determinants are operationalized in various ways. We suggest for future research to bring more focus to which behavior, which determinant, and which facet of SES are studied. For example, future research should consider the various operationalizations of SES, such as (parental) education, income, work, and neighborhood and confounding factors like minority status, race/ethnicity, and area of residence (e.g., urban vs. rural), that may have varying effects on obesity in childhood ([6, 7]; Ogden et al., 2010, in [8]).

## 6. Conclusions

Our review demonstrates that there is very little research done regarding the role of SES on modifying the impact of determinants on obesity and obesity-related lifestyle behaviors for children 0–12 years of age. For example, for the social–cognitive determinants of the theory of planned behavior [19] and the peer and school determinants, we did not find research focusing on 0- to 12-year-old children. The results of the few existing systematic reviews/meta-analyses provided scarce and sometimes inconsistent evidence. In addition, the multifaceted nature of SES might contribute to this inconsistency of findings in the literature. Moreover, the underlying theoretical explanations/mechanisms through which SES has its influence on child obesity are still unclear. There is a lack of theories why determinants may have an effect on obesity and obesity-related lifestyle behaviors. Very often, the systematic reviews/meta-analyses only describe the effect of a diverse set of determinants on obesity-related behaviors like the neighborhood determinants. Furthermore, the majority of the existing studies included in the systematic reviews/meta-analyses conducted cross-sectional studies, and thus, cause–effect conclusions cannot be drawn from these findings. In order to develop effective (family) interventions to prevent or decrease obesity in children from low SES families, we first need to gain more insights into the determinants and thus the underlying theoretical explanations/mechanisms through which SES has its influence on child obesity.

## Figures and Tables

**Table 1:** Description of the included reviews of obesity and obesity-related lifestyle behaviors.

Review	Type of review	Number of studies	Type of studies	Sample	Role of SES^a^	Level^b^
Anzman-Frasca et al. [26]	Systematic review	18^c^	Cross-sectional and longitudinal	Children		Individual
Anderson et al. [63]	Systematic review	28	Cross-sectional (except 1 prospective)	Children and adolescents (0–18 years)	X	Community (neighborhood)
Benton et al. [40]	Systematic review	20	Cross-sectional and longitudinal	Children		Interpersonal (family/parents)
Bingham et al. [47]	Systematic review	130	Cross-sectional	Children		Interpersonal (family/parents)
Blaine et al. [50]	Systematic review	47	Cross-sectional, longitudinal, and experimental	Children and adolescents (2–18 years)		Interpersonal (family/parents)
Clark et al. [42]	Systematic review	26	Observational, qualitative, cross-sectional, experimental, retrospective, and longitudinal	Children		Interpersonal (family/parents)
Dallacker et al. [45]	Meta-analysis	50	Cross-sectional and longitudinal	Children	X	Interpersonal (family/parents)
Davison and Lawson [61]	Systematic review	33	Longitudinal, cross-sectional, and intervention	Children and adolescents (3–18 years)		Community (neighborhood)
Dunton et al. [59]	Systematic review	15	Cross-sectional, quasiexperimental, and longitudinal	Children	X	Community (neighborhood)
Faith et al. [43]	Systematic review	22	Cross-sectional and longitudinal	Children		Interpersonal (family/parents)
Francis et al. [58]	Systematic review	17	Longitudinal, cross-sectional, and observational, in-depth interviews, focus groups	Children (2–5 years) at family child care homes		Organizational (school)
Gebremariam et al. [51]	Systematic review	37	Cross-sectional and longitudinal	Children and adolescents (≤ 18)	X	Interpersonal (family/parent)
Gebremariam et al. [37]	Systematic review	28	Cross-sectional and longitudinal	Children and adolescents (≤ 18)	X	Interpersonal (family/parent)
Gray et al. [57]	Systematic review	12	Longitudinal	Children (4–15 years)		Organizational (school)
Hagger and Chatzisarantis [29]	Meta-analysis	34	Cross-sectional, experimental, intervention	Children, adolescents, and adults		Individual
Hails et al. [28]	Systematic review	25	Longitudinal	Children	X^d^	Individual
Halliday et al. [41]	Systematic review	21	Cross-sectional and longitudinal	Children and adolescents		Interpersonal (family/parents)
Incledon et al. [27]	Systematic review	15	Longitudinal	Children		Individual
Kaushal and Rhodes [54]	Systematic review	49	Experimental and observational	Children		Organizational (physical home)
Kiefner-Burmeister and Hinman [32]	Systematic review	24	Longitudinal, cross-sectional, prospective, experimental	Children and adolescents		Interpersonal (family/parents)
Kim et al. [60]	Systematic review	39	Cross-sectional and longitudinal	Children (3–17 years)		Community (neighborhood)
Kininmonth et al. [46]	Systematic review	62	Cross-sectional, prospective	Children		Interpersonal (family/parents) and organizational (physical home)
Lampard et al. [39]	Systematic review	9	Longitudinal	Children	X^e^	Interpersonal (family/parents)
Lindsay et al. [11]	Systematic review	158	Quantitative and qualitative	Children		Interpersonal (family/parent), Community(neighborhood), and societal (culture)
Maitland et al. [55]	Systematic review	49	Observational and experimental	Children (8–14 years)		Organizational (physical home)
Mamrot and Hanć [25]	Systematic review	27	Cross-sectional and longitudinal	Children and adolescents		Individual
Maturo and Cunningham [52]	Systematic review	81	Cross-sectional and longitudinal	Children and adolescents		Interpersonal (peers)
McClain et al. [31]	Systematic review	77	Cross-sectional and prospective	Children and adolescents		Individual
Mech et al. [36]	Systematic review	30	Cross-sectional and longitudinal	Children	X	Interpersonal (family/parents)
Oken et al. [38]	Meta-analysis	14	Observational	Children	X	Interpersonal (family/parent)
Rageliene and Grønhøj [53]	Systematic review	29	Cross-sectional, longitudinal, experimental, focus groups, and interviews	Children and adolescents		Interpersonal (peers)
Sallis et al. [30]	Systematic review	54	Cross-sectional and prospective	Children		Individual
Sanchez‐Vaznaugh et al. [65]	Systematic review	18	Cross-sectional, pre–post design	Children and adolescents		Societal (policy)
Shloim et al. [33]	Systematic review	31	Cross-sectional, longitudinal, and experimental	Children		Interpersonal (family/parent)
Sleddens et al. [34]	Systematic review	36	Cross-sectional and longitudinal	Children		Interpersonal (family/parent)
Smith et al. [62]	Systematic review	28	Cross-sectional, case–control, and longitudinal	Children		Community (neighborhood)
Valdes et al. [44]	Systematic review	15	Cross-sectional and longitudinal	Children		Interpersonal (family/parent)
Vollmer and Mobley [35]	Systematic review	51	Cross-sectional, longitudinal, prospective, case–control	Children		Interpersonal (family/parents)
Yao and Rhodes [48]	Meta-analysis	115	Cross-sectional and prospective	Children		Interpersonal (family/parents)
Yee et al. [49]	Meta-analysis	37	Cross-sectional, longitudinal, experimental, and quasiexperimental	Children and adolescents		Interpersonal (family/parents)
Zhang et al. [56]	Systematic review	8	Cross-sectional, longitudinal, and intervention	Children (6 years)		Organizational (school)
Zhang et al. [64]	Systematic review	21	Cross-sectional and longitudinal	Children (< 18 years) of immigrants		Societal (culture)

^a^Role of SES refers to tested indirect relation and/or moderation effect.

^b^Level refers to the individual or environmental (i.e., interpersonal, organizational, community, societal) level of the determinant.

^c^Number of studies included in the systemic review/meta-analysis.

^d^SES was included as poverty.

^e^SES was included as income.

**Table 2:** Results at the individual level of the included reviews of obesity and obesity-related lifestyle behaviors (i.e., eating behavior, physical activity, sedentary behavior).

Determinant	Outcome	+	0	−	?	Role of SES: indirect relation	Role of SES: moderation effect
*Individual level*							
Inhibitory control	Obesity indicators			[25]		n.a.	n.a.
Self-regulation	Overweight/obesity			[26]		Yes poverty [28]	n.a.
Temperamental negative reactivity	Overweight	[26]				n.a.	n.a.
Low/poor self-esteem (SE) (e.g., physical appearance self-esteem, global self-esteem)	Obesity/overweight	Reference [27] [poor global SE]	Reference [27] [SE in overweight children]		Reference [27] [physical appearance SE]	n.a.	n.a.
Intention	Physical activity	References [29, 30]				n.a.	n.a.
Perceived barriers	Physical activity			[30]		n.a.	n.a.
Preferences	Physical activity	[30]				n.a.	n.a.
Self-efficacy	Physical activity				[30]	n.a.	n.a.
Attitudes	Physical activity				[30]	n.a.	n.a.
Intention	Eating healthy	Reference [31] [intention to eat healthy/outcome: Fruit, juice, and vegetable intake]		Reference [31] [intention to consume sugar/outcome: Sugar snacking]		n.a.	n.a.
Preferences	Eating healthy	Reference [31] [fruit, juice, and vegetable intake]				n.a.	n.a.
Liking	Eating healthy	Reference [31] [fruit, juice, and vegetable intake]				n.a.	n.a.

*Note:* +, positive association; 0, no association; −, negative association; ?, mixed results.

Abbreviation: SES, socioeconomic status.

**Table 3:** Results at the environmental level of the included reviews of obesity and obesity-related lifestyle behaviors (i.e., eating behavior, physical activity, sedentary behavior).

Determinant	Outcome	+	0	−	?	Role of SES: indirect relation	Role of SES: moderation effect
*Environmental level*							
Interpersonal level							
Family/parents							
Smoking during pregnancy	Overweight/adiposity	[38]				No SES [36, 38] Yes SES^a^ [37]	n.a.
Mother's depression	(Over)weight	Reference [39] [chronic depression] [40];	Reference [39] [episodic depression]			No SES [36]	Yes SES [36] No income ([39] [chronic depression])
Parental BMI	Overweight/adiposity					Yes SES [36, 37]	Yes SES [36]
Permissive parenting style	Overweight/BMI	[35]				n.a.	Yes SES [36]
Authoritative parenting style (i.e., warm and demanding parenting)	BMI			Reference [32–35]		n.a.	n.a.
Authoritarian parenting style	BMI	[35]				n.a.	n.a.
Feeding style (emotional feeding, instrumental feeding)	Overweight		[43]			No SES [36]	n.a.
Authoritative feeding style	BMI			Reference [33, 35]		n.a.	n.a.
Indulgent feeding style	BMI	Reference [33, 35]				n.a.	n.a.
Restrictive/controlling feeding	Obesity/(over)weight	Reference [33, 42, 43]				n.a.	n.a.
Pressure to eat enough food	BMI			[33]		n.a.	n.a.
Monitoring child intake	BMI		[33]			n.a.	n.a.
Family functioning (e.g., poor communication, poor behavior control, high levels of family conflict, low family hierarchy values)	Overweight/obesity	[41]				n.a.	n.a.
Frequency of family meals	Overweight/BMI			[45]	[44]	n.a.	No SES [45]
Caregiver modeling/support of food intake	Adiposity/BMI				[46]		
Caregiver rules and limit setting around unhealthy eating	Adiposity/BMI		[46]				
Caregiver modeling/support of physical activity	Adiposity/BMI				[46]		
Caregiver modeling of media use	Adiposity/BMI				[46]		
Caregiver rules and limit setting around media use	Adiposity/BMI				[46]		
Having a TV in the child's bedroom	Sedentary behavior					Yes SES [51]	n.a.
Parental modeling for TV viewing	Sedentary behavior					Yes SES [51]	n.a.
Parental co-viewing	Sedentary behavior					Yes SES [51]	n.a.
Eating meals in front of the TV	Sedentary behavior					Yes SES [51]	n.a.
Rules and regulations about screen time	Sedentary behavior					No SES [51]	n.a.
Parents' beliefs that participating in PA is important	Physical activity (PA)	[11]				n.a.	n.a.
Parents' positive attitudes toward PA	Physical activity	[11]				n.a.	n.a.
Parents' concerns about safety (including concerns related to neighborhood and community safety such as crime and traffic)	Physical activity			[11]		n.a.	n.a.
Encouraging PA	Physical activity	[11]				n.a.	n.a.
Setting rules	Physical activity	[11]				n.a.	n.a.
Parental support	Physical activity	Reference [47, 48]				n.a.	n.a.
Authoritative parenting style (i.e., warm and demanding parenting)	Physical activity	[34]			[35]	n.a.	n.a.
Engaging in PA activities with children/time spent playing with parents	Physical activity	Reference [11, 47]				n.a.	n.a.
Parental modeling of PA/parents' physical activity	Physical activity	Reference [11, 47, 48]				n.a.	n.a.
Providing transportation, paying fees and tuition	Physical activity	[11]				n.a.	n.a.
Authoritative parenting style (i.e., warm and demanding parenting)	Healthier diet	Reference [32, 34, 35]				n.a.	n.a.
Authoritative feeding style	Healthier diet		[35]			n.a.	n.a.
Parental restriction of food	Snack intake/eating	Reference [43, 50]				n.a.	n.a.
Parental control of availability of unhealthy foods	Eating unhealthy			[49]		n.a.	n.a.
Pressure to eat	Snack intake				[50]	n.a.	n.a.
Monitoring food intake	Snack intake				[50]	n.a.	n.a.
Home availability of unhealthy foods	Snack intake	[50]				n.a.	n.a.
Nonavailability of unhealthy foods	Eating unhealthy			[49]		n.a.	n.a.
Availability of healthy foods	Eating healthy	[49]				n.a.	n.a.
Availability of unhealthy foods	Eating unhealthy	[49]				n.a.	n.a.
Positive parent behaviors (i.e., role modeling and rules about eating)	Snack intake				[50]	n.a.	n.a.
Frequency in which parents eat healthily (i.e., parental modeling)	Eating healthy	[49]				n.a.	n.a.
Unhealthy parent eating behaviors (i.e., role modeling and rules about eating)	Eating unhealthy	[49]				n.a.	n.a.
Norms	Eating healthy	Reference [31] [fruit, juice, and vegetable intake]		Reference [31] [sweetened beverage consumption]		n.a.	n.a.
Perceived modeling	Eating healthy	Reference [31] [fruit, juice, and vegetable intake; child report]		Reference [31] [sweetened beverage consumption; child report]	Reference [31] [sweetened beverage consumption; fruit, juice, and vegetable intake; parent report]	n.a.	n.a.
Peers							
Encouragement from friends (i.e., communicating social norms)	Physical activity	[52]				n.a.	n.a.
Friends' own PA (i.e., modeling)	Physical activity	[52]				n.a.	n.a.
Engagement with friends in PA	Physical activity	[52]				n.a.	n.a.
Peers' liking of unhealthy food	Eating healthy			[53]		n.a.	n.a.
Friends' concern for eating healthy food	Eating healthy	[53]				n.a.	n.a.
Peer support for unhealthy eating	Eating healthy			[53]		n.a.	n.a.
Eating out with peers	Eating healthy			[53]		n.a.	n.a.
Adaptation to the eating habits of peers	Eating healthy			[53]		n.a.	n.a.
Peer approval	Eating healthy	[53]				n.a.	n.a.
Descriptive norms among peers	Eating healthy	[53]				n.a.	n.a.
Perceived engagement level of siblings	Eating healthy	[53]				n.a.	n.a.
Organizational level							
Physical home							
Food availability/access in the home	Adiposity/BMI				[46]		
Physical activity equipment availability/access in the home	Adiposity/BMI				[46]		
Media equipment availability/access (e.g., TV, computer, mobile phone) in the home/bedroom	Adiposity/BMI	[46]					
TV limiting devices	Sedentary behavior (i.e., television time)			Reference [54, 55]		n.a.	n.a.
Media equipment (e.g., TV, computer, mobile phone) in the home/bedroom	Sedentary behavior	Reference [54, 55]				n.a.	n.a.
Physical activity equipment	Sedentary behavior			[55]		n.a.	n.a.
Using prominent exergaming materials (i.e., exergaming bike, dance mats)	PA	[54]				n.a.	n.a.
Presence of a backyard	PA	[54]				n.a.	n.a.
The house (e.g., size, space, design) and yard	PA		[55]			n.a.	n.a.
Presence of PA materials/equipment	PA	[54]			[55]	n.a.	n.a.
Active videogames	PA				[55]	n.a.	n.a.
School							
Indoor space for active play	Healthy weight	[56]				n.a.	n.a.
Portable and fixed play equipment	Healthy weight	[56]				n.a.	n.a.
Lack of adequate equipment and space for indoor and outdoor playtime activities	Obesity	[58]				n.a.	n.a.
Time of free play outdoor and indoor	Healthy weight	[56]				n.a.	n.a.
Sedentary opportunities (e.g., seated activities, TV viewing, and video game playing)	Healthy weight			[56]		n.a.	n.a.
No high sugar and high fat food served	Healthy weight	[56]				n.a.	n.a.
Poor feeding practices	Obesity	[58]				n.a.	n.a.
Healthful school food environment	Obesity			[57]		n.a.	n.a.
Lack of nutrition and physical activity policies	Obesity	[58]				n.a.	n.a.
School's SES index score	Obesity			[57]		n.a.	n.a.
Having healthy-weight educators and active educators	Healthy weight	[56]				n.a.	n.a.
Lack of providers knowing effective nutrition and physical activities	Obesity	[58]				n.a.	n.a.
Misperceptions regarding nutrition among providers	Obesity	[58]				n.a.	n.a.
Minutes of recess and physical education	Obesity			[57]		n.a.	n.a.
Meeting recommended recess and physical education time	Obesity			[57]		n.a.	n.a.
Poor nutrition-related communication with families	Obesity	[58]				n.a.	n.a.
Percentage of parental involvement in school	Obesity			[57]		n.a.	n.a.
Community level							
Neighborhood							
Neighborhood economic status	Obesity			[60]		n.a.	n.a.
Road safety	Obesity			[59]		n.a.	n.a.
Neighborhood hazards (e.g., litter, trash, noise)	BMI	[59]				n.a.	Yes SES [59]
School play space	Obesity			[59]		n.a.	n.a.
Proximity to supermarkets	Obesity			[59]		n.a.	n.a.
Neighborhood economic status	PA				[60]	n.a.	n.a.
Built environment (accessibility, safety, comfort, and pleasurability)	PA					No SES [63]	No SES [63] [except for accessibility]
Publicly provided recreational infrastructure (access to recreational facilities and schools)	PA	[61]				n.a.	n.a.
Publicly provided transport infrastructure (e.g., presence of sidewalks and controlled intersections, access to destinations and public transportation)	PA	Reference [61, 62]				n.a.	n.a.
Transport infrastructure (number of roads to cross and traffic density/speed)	PA			[61]		n.a.	n.a.
Local socioeconomic conditions (crime, area deprivation; e.g., rates of car ownership, housing tenure, unemployment, and overcrowding in the district)	PA			[61]		n.a.	n.a.
Perceived neighborhood safety	PA		[61]			n.a.	n.a.
Perceived lack of neighborhood safety	PA			[11]		n.a.	n.a.
Age appropriate, outdoor play spaces with access to play equipment	PA	Reference [11, 62]				n.a.	n.a.
Neighborhood economic status	Eating				[60]	n.a.	n.a.
Societal level							
Culture							
Acculturation	Body weight				[64]	n.a.	n.a.
Acculturation	PA				[11]	n.a.	n.a.
Acculturation	Dietary intake				[64]	n.a.	n.a.
Policy							
Policies to regulate competitive foods and beverages (e.g., candy, chips, and sodas) sold in schools	Overweight/obesity			[65]			

*Note:* +, positive association; 0, no association; −, negative association; ?, mixed results.

Abbreviation: SES, socioeconomic status.

^a^SES operationalized as income per capita, education (maternal), income deprivation, poverty (trajectory), composite scores, or occupation.

**Table 4:** Scale for the assessment of narrative review articles-SANRA.

Checklist item	Reported on page
1. Justification of the article's importance for the readership	3–4
2. Statement of concrete aims or formulation of questions	4
3. Description of the literature search	4–5
4. Referencing	6–15
5. Scientific reasoning	6–15 & Table 1
6. Presentation of data	6–11 & Tables 2 and 3

**Table 5:** Narrative review checklist.

Section/topic	#	Checklist item	Reported on page or line #
*Title*			
Title	1	Identify the report as a narrative review of…	1

*Abstract*			
Unstructured summary	2	Provide an unstructured summary including as applicable: background, objective, brief summary of narrative review and implications for future research, and clinical practice or policy development.	2

*Introduction*			
Rational/background	3	Describe the rationale for the review in the context of what is already known.	3–4
Objectives	4	Specify the key question(s) identified for the review topic.	4

*Methods*			
Research selection	5	Specify the process for identifying the literature search (eg. years considered, language, publication status, study design, and databases of coverage).	4–5

Discussion/summary			
Narrative	6	Discuss: (1) research reviewed including fundamental or key findings, (2) limitations and/or quality of research reviewed, and (3) need for future research.	11–13
Summary	7	Provide an overall interpretation of the narrative review for health professionals, policy development and implementation, or future research.	13–15

## Data Availability

All publications used in the work are available in PsycINFO/Ovid, PubMed, and online.
